# Supplementary material for: Subtractive transformation of cathode materials in spent Li-ion batteries to a low-cobalt 5 V-class cathode material
Source: Nat Commun. 2024 Feb 5;15:1046. doi: 10.1038/s41467-024-45091-8 (PMC10844610; doi:10.1038/s41467-024-45091-8)
Supplement: Supplementary file 1 — Supplementary Information [file 41467_2024_45091_MOESM1_ESM.pdf]

Supplementary Information

Subtractive transformation of cathode materials in spent Li-ion batteries to a low-cobalt 5 V-class cathode material

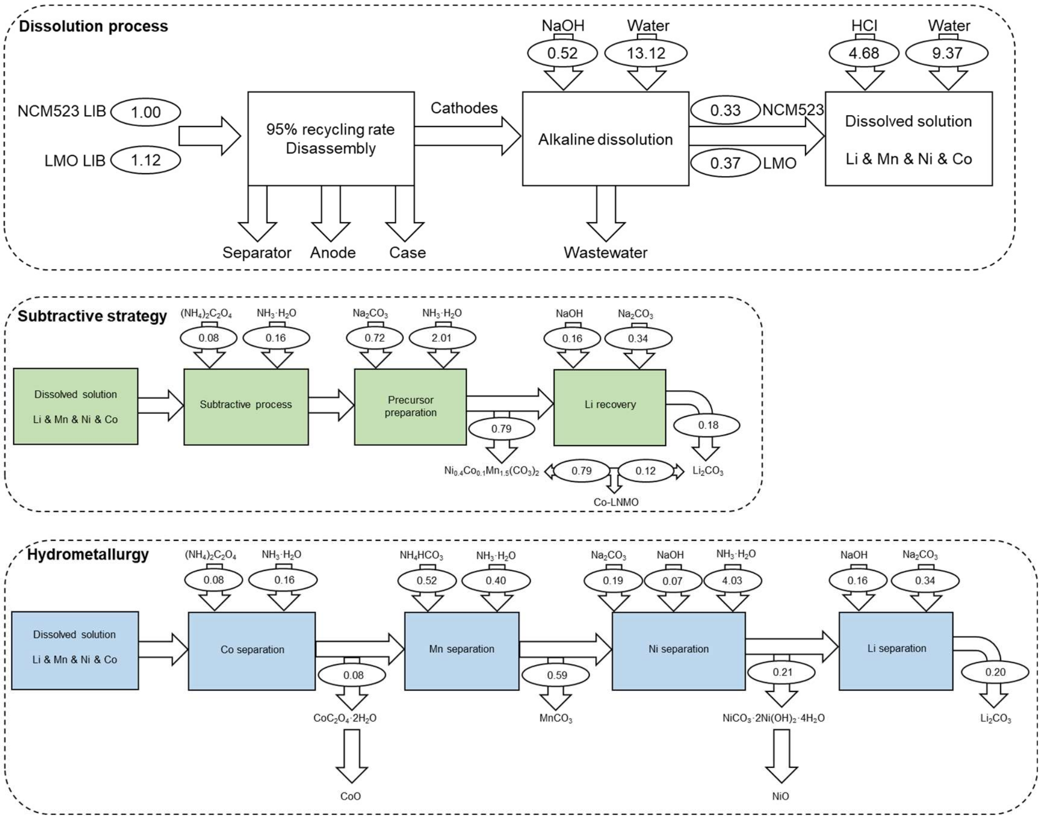

Supplementary Fig. 1 The dissolution process for spent cathode materials and the comparison of the subtractive strategy and the regular hydrometallurgy strategy. Suppose that 1 ton of ternary NCM523 spent batteries are treated and the regular hydrometallurgy strategy uses the common and inexpensive inorganic extractants, that the recovery rate is 100%, and that the recovered products are all transformed into commercial products without further purification. The processing steps involved in the subtractive strategy are fewer in contrast to the multi-step separation processes in the regular hydrometallurgy strategy.

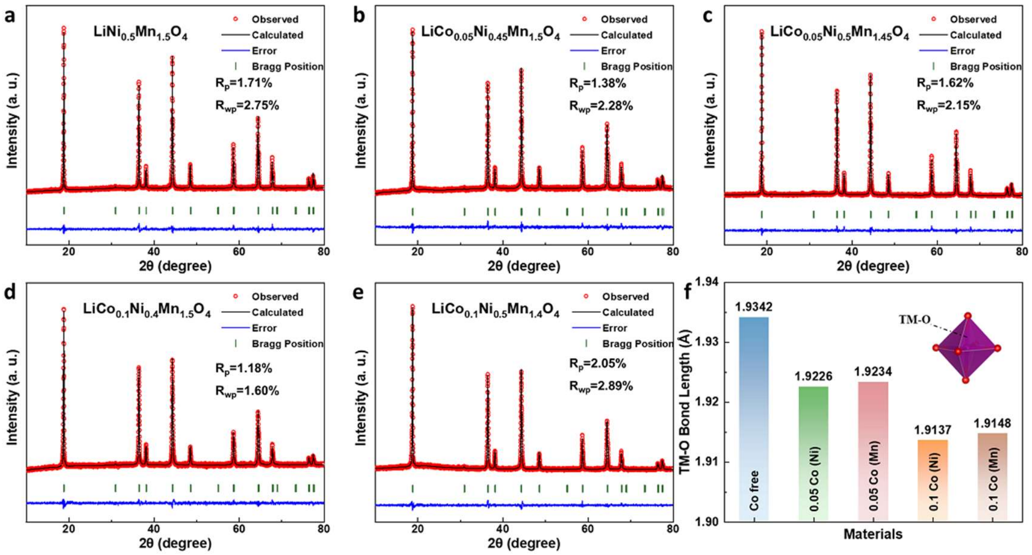

Supplementary Fig. 2 a-e, Rietveld refinement of $LiNi_{0.5}Mn_{1.5}O_4$ **(a)**, $LiCo_{0.05}Ni_{0.45}Mn_{1.5}O_4$ **(b)**, $LiCo_{0.05}Ni_{0.5}Mn_{1.45}O_4$ **(c)**, $LiCo_{0.1}Ni_{0.4}Mn_{1.5}O_4$ **(d)**, $LiCo_{0.1}Ni_{0.5}Mn_{1.4}O_4$ **(e)** from XRD data. **f,** transition metal-oxygen (TM-O) bond length of LNMO samples with 0.05/0.1-mole ratios Co substitution of Ni or Mn based on the Rietveld refinement results.

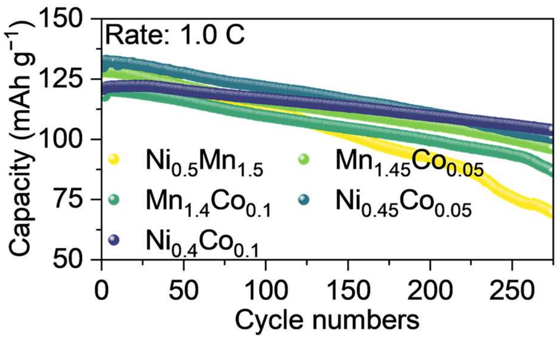

Supplementary Fig. 3 Discharge capacities of LNMO samples with 0.05/0.1-mole ratios Co substitution of Ni or Mn or not during cycling 1 C rate at 30°C.

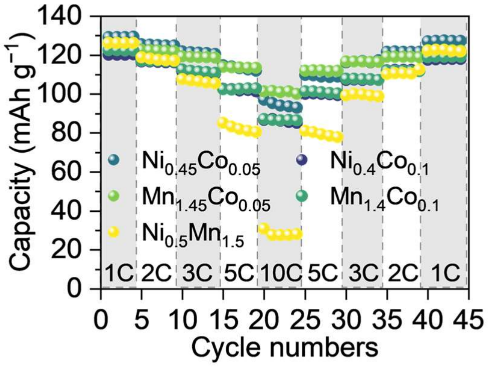

Supplementary Fig. 4 Galvanostatic rate capability measurements of LNMO samples with 0.05/0.1-mole ratios Co substitution of Ni or Mn or not during cycling at 30℃ in a constant-current (CC) mode, 1 C is equal to 146 mAh·g^{-1}.

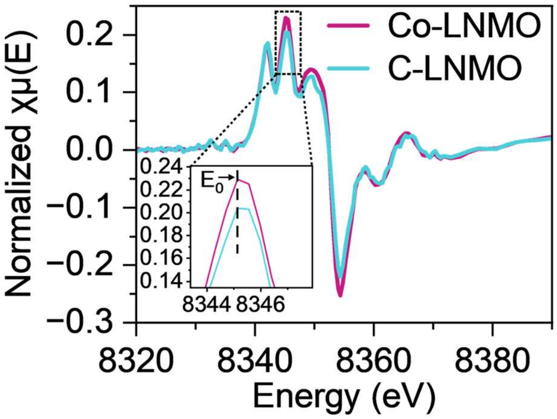

Supplementary Fig. 5 The first derivative of XANES of Co-LNMO and C-LNMO samples (E_0 shown in the inset).

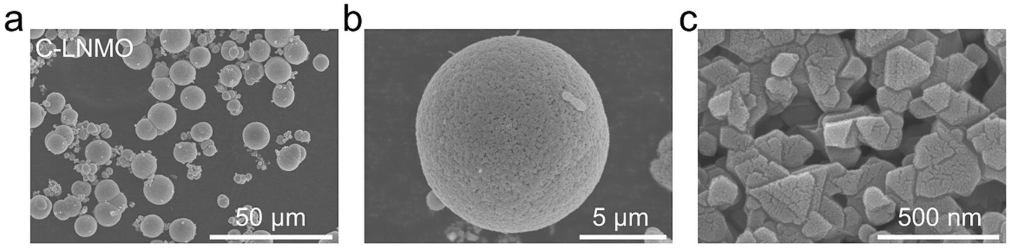

Supplementary Fig. 6 a,b, SEM images of C-LNMO with secondary particle structure (50 μm scale bar) **(a)** and (5 μm scale bar) **(b)**. **c,** SEM image of a primary particle of C-LNMO (500 nm scale bar).

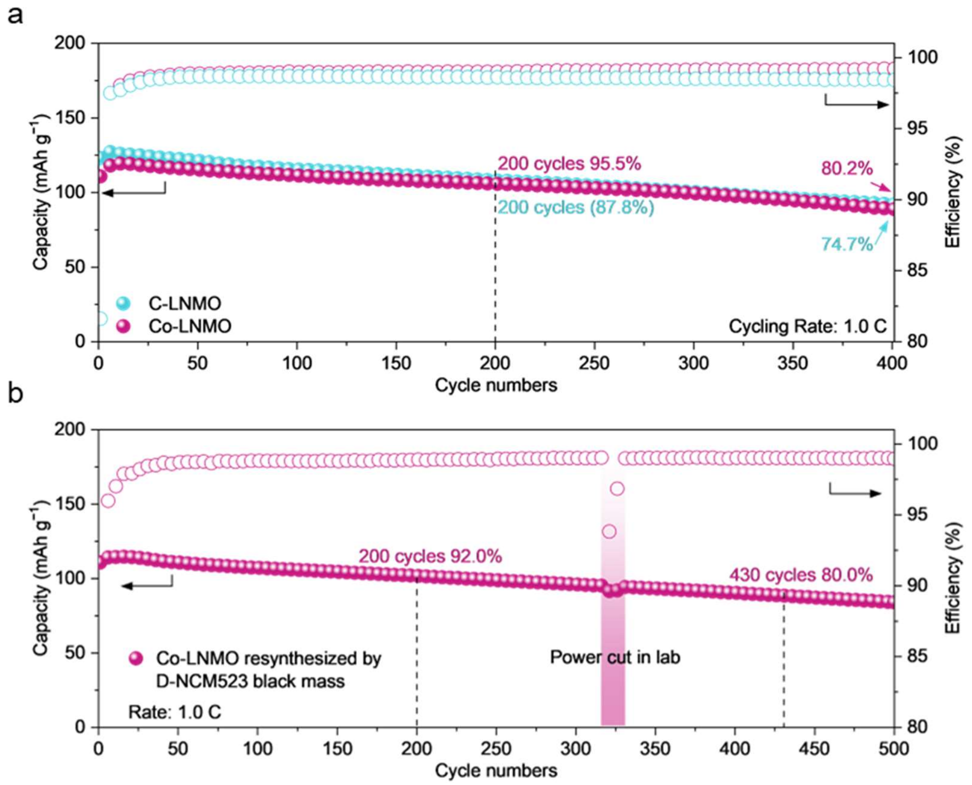

Supplementary Fig. 7 a, Cycling performance of Co-LNMO and C-LNMO under 1 C within 3.0-4.95 V (vs. Li+/Li) in half cells. **b,** Discharge capacities of Co-LNMO resynthesized by D-NCM523 black mass during cycling 1 C rate at 30°C for 500 cycles.

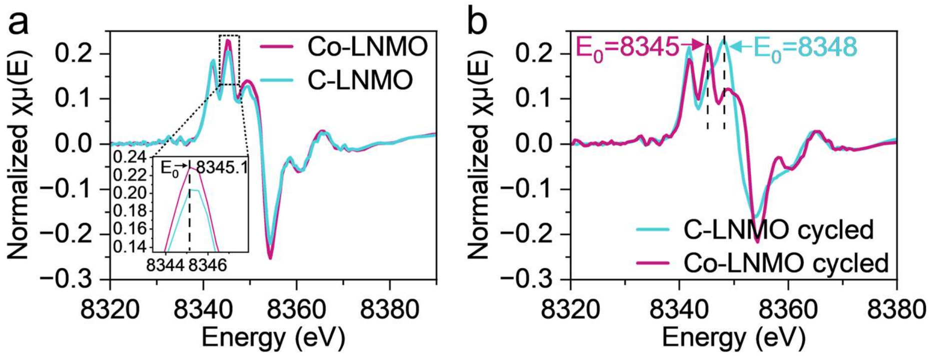

Supplementary Fig. 8 a,b, The first derivative of XANES of pristine **(a)** and cycled **(b)** Co-LNMO and C-LNMO samples (E_0 shown in the inset).

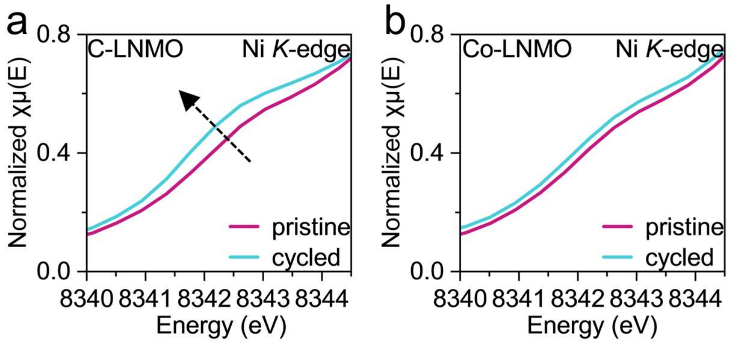

Supplementary Fig. 9 a,b, The Ni K-edge XANES spectrum at 8340 to 8344 eV of pristine and cycled C-LNMO **(a)** and Co-LNMO **(b)**.

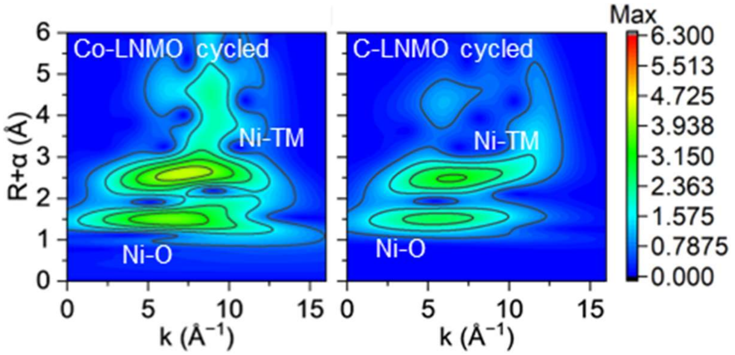

Supplementary Fig. 10 The 2D contour Fourier-transformed Ni K-edge EXAFS analysis of cycled Co-LNMO and C-LNMO.

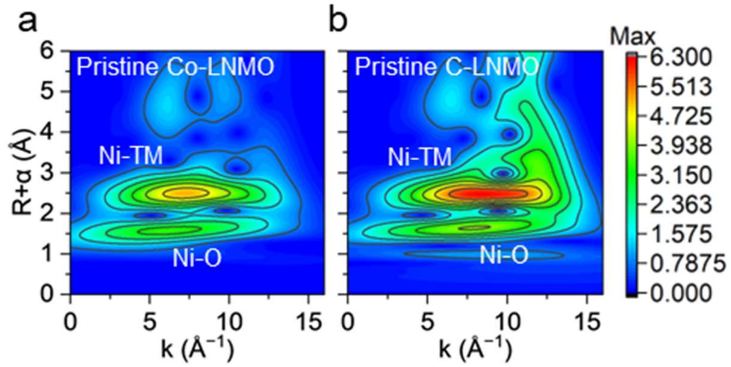

Supplementary Fig. 11 a,b, The 2D contour Fourier-transformed Ni K-edge EXAFS analysis of pristine Co-LNMO **(a)** and pristine C-LNMO **(b)**.

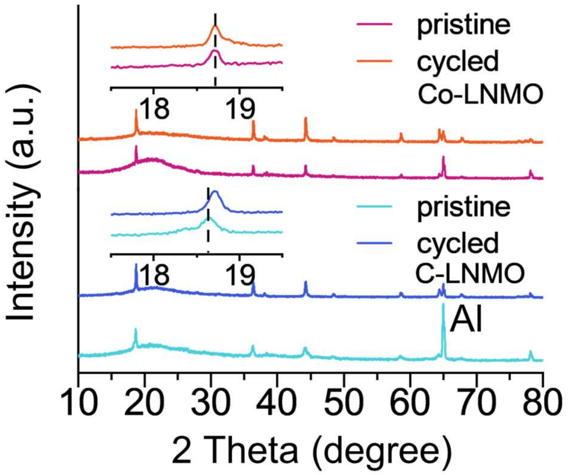

Supplementary Fig. 12 XRD patterns and characteristic peak differences of pristine and cycled Co-LNMO and C-LNMO.

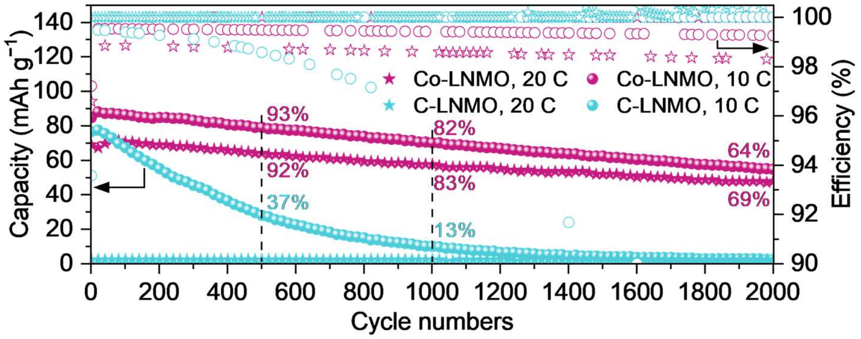

Supplementary Fig. 13 Discharge capacities of Co-LNMO and C-LNMO during cycling 10 C rate and 20 C at 30°C for 2000 cycles.

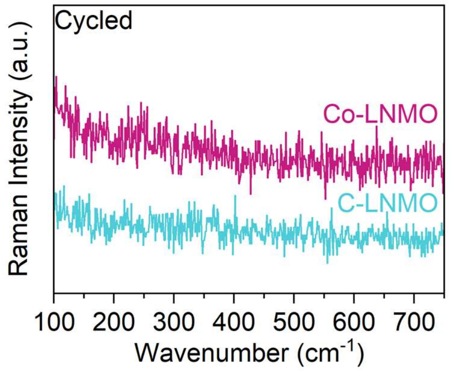

Supplementary Fig. 14 Post-mortem Raman spectra of Co-LNMO and C-LNMO cathodes harvested from cycled cells.

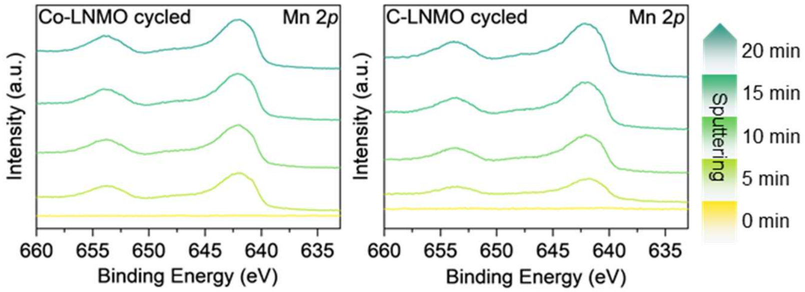

Supplementary Fig. 15 XPS spectra of Mn 2*p* at different depths of Co-LNMO and C-LNMO after 400th cycling at 1 C rate, the acquisition of signal was collected every 5 min by Ar^+ sputtering.

Supplementary Table 1 Comparison of regular hydrometallurgical recycling methods from the reported works

Cathode material	Reagent	Product	Recovery rate
NCM [1]	H_2SO_4 and H_2O_2, DMG ($C_4H_8N_2O_2$), P204 and C272	Ni-$(C_4H_8N_2O_2)_2$, MnO_2, CoC_2O_4 and Li_2CO_3	Ni (96.84%), Co (81.46%), and Mn (92.65%)
NCA [2]	H_2SO_4, H_2O_2 D2EHPA-Bis(2-ethylhexyl) phosphoric acid, Cyanex272®-Bis 2,4,4-trimethylpentyl phosphinic acid	Co_3O_4 (purity 83%), CoC_2O_4 (purity 96%), NiO (purity 89%), and Li_2CO_3 (purity 99.8%)	Co (80%~85%), Ni (90%) Li (72%).
NCM [3]	Reduction roasting with starch, H_2SO_4, Mextral 984H (5-nonylsalicylaldoxime and 2-hydroxy-5-nonylacetophenone oxime),	No	Co (90.9%), Li (82%), Ni and Cu (98%)
NCM [4]	$KMnO_4$, IL (trihexyltetradecylphosphonium bis(2,4,4-trimethylpentyl)phosphinate),	split-phosphinate molecule (as CoA_2) and stabilization of phosphonium	extraction equilibria of Co^{2+} with ionic liquid (>99%)

	aromatic solvent C10, NaOH	molecule by complexing with the chloride ions of aqueous solution (as $R_4P^+Cl^-$) of the used ionic liquid (i.e., $R_4P^+A^-$).	
NCA [5]	HCl, NaClO, NaOH	$Co_2O_3 \cdot 3H_2O$, $Ni(OH)_2$	Co (90.25%) and Ni (96.36%), Li (>80%)
NCM [6]	HCl, hypochlorite solution (NaClO), Na_2CO_3	Mixture of manganese oxides (MnO_2, Mn_3O_4, $Na_{0.55}Mn_2O_4 \cdot 1.5 H_2O$, $CoCO_3$, and Li_2CO_3	Mn (95%) and Co (90%)
NCM [7]	NH_3, $(NH_4)_2SO_4$, Na_2SO_3	$(NH_4)_2Mn(SO_3)_2 \cdot H_2O$, Li_2SO_4, amine complexes	NA, Leaching rate: Ni (94.8%), Co (88.4%), Mn (6.34%), and Li (96.7%)
NCM [8]	$(NH_4)_2SO_4$, $(NH_4)_2SO_3$	Li_2SO_4,	NA, Leaching

		$(NH_4)_2Co(SO_4)_2\cdot H_2O$, $(NH_4)_2Mn(SO_3)_2\cdot H_2O$ and $(NH_4)_2Mn(SO_4)_2\cdot 6H_2O$	rate: Ni (98%), Co (81%), Mn (92%), and Li (98%)

Supplementary Table 2 Refinement results of re-synthesized Co-LNMO and C-LNMO.

Co-LNMO Refinement Results

**Co-LNMO** (Space Group Fd-3m)						
	**Atom**	**x**	**y**	**z**	**Occ.**	**Biso.**
**Atomic Occupancies**	Li	0.1250	0.1250	0.1250	1.0	1.1327(2)
	Ni	0.5000	0.5000	0.5000	0.20(2)	
	Mn	0.5000	0.5000	0.5000	0.74(9)	1.0930(4)
	Co	0.5000	0.5000	0.5000	0.04(9)	
	O	0.2638	0.2638	0.2605(3)	1.0	0.7777(4)
**Lattice Parameters**	***a* / Å (*a*=*b*=*c*)**			**V / Å^3**		
	8.1637(1)			544.07(9)		
**Agreement Factors**						
χ^2	3.16%		R_p 2.04%		R_{wp} 2.66%	

C-LNMO Refinement Results

**LNMO** (Space Group Fd-3m)						
	**Atom**	**x**	**y**	**z**	**Occ.**	**Biso.**
**Atomic Occupancies**	Li	0.1250	0.1250	0.1250	1.0	1.5979(2)
	Ni	0.5000	0.5000	0.5000	0.25(1)	1.1548(5)
	Mn	0.5000	0.5000	0.5000	0.74(9)	
	O	0.2638	0.2638	0.2611(7)	1.0	0.7370(4)
**Lattice Parameters**	***a* / Å (*a*=*b*=*c*)**			**V / Å^3**		
	8.1656(6)			544.47(1)		
**Agreement Factors**						
χ^2	3.35%		R_p 2.16%		R_{wp} 2.83%	

References

1. Li, C. *et al*. Separation and recovery of nickel cobalt manganese lithium from waste ternary lithium-ion batteries. *Separation and Purification Technology* **306**, 122559 (2023)

2. Atia, T. A., Elia, G., Hahn, R., Altimari, P. & Pagnanelli, F. Closed-loop hydrometallurgical treatment of end-of-life lithium ion batteries: Towards zero-waste process and metal recycling in advanced batteries. *Journal of Energy Chemistry* **35**, 220 (2019).

3. Yang, C., Zhang, J., Liang, G., Jin, H., Chen, Y. & Wang, C. An advanced strategy of "metallurgy before sorting" for recycling spent entire ternary lithium-ion batteries. *Journal of Cleaner Production* **361**, 132268 (2022).

4. Ilyas, S., Srivastava, R. R. & Kim, H. Selective separation of cobalt versus nickel by split-phosphinate complexation using a phosphonium-based ionic liquid. *Environmental Chemistry Letters* **21**, 673 (2023).

5. Joulié, M., Laucournet, R. & Billy, E. Hydrometallurgical process for the recovery of high value metals from spent lithium nickel cobalt aluminum oxide based lithium-ion batteries. *Journal of Power Sources* **247**, 551-555 (2014).

6. Barik, S., Prabaharan, G. & Kumar, L. Leaching and separation of Co and Mn from electrode materials of spent lithium-ion batteries using hydrochloric acid: Laboratory and pilot scale study. *Journal of Cleaner Production* **147**, 37-43 (2017).

7. Zheng, X. *et al*. Spent lithium-ion battery recycling–Reductive ammonia leaching of metals from cathode scrap by sodium sulphite. *Waste Management* **60**, 680-688 (2017).

8. Chen, Y., Liu, N., Hu, F., Ye, L., Xi, Y. & Yang, S. Thermal treatment and ammoniacal leaching for the recovery of valuable metals from spent lithium-ion batteries. *Waste Management* **75**, 469-476 (2018).
